# Arsenic Trioxide and Thalidomide Combination Induces Autophagy Along with Apoptosis in Acute Myeloid Cell Lines

**DOI:** 10.22074/cellj.2020.6469

**Published:** 2019-10-14

**Authors:** Mahnaz Mohammadi Kian, Atousa Haghi, Mahdieh Salami, Bahram Chahardouli, Shahrbanoo Rostami, Kianoosh Malekzadeh, Hosein Kamranzadeh Foumani, Saeed Mohammadi, Mohsen Nikbakht

**Affiliations:** 1. Hematology, Oncology and Stem Cell Transplantation Research Center, Tehran University of Medical Sciences, Tehran, Iran; 2. Hematologic Malignancies Research Center, Tehran University of Medical Sciences, Tehran, Iran; 3. Young Researchers and Elite Club, Pharmaceutical Sciences Branch, Islamic Azad University, Tehran, Iran; 4. Molecular Medicine Research Center (MMRC), Hormozgan University of Medical Science (HUMS), Bandar Abbass, Iran

**Keywords:** Acute Myeloid Leukemia, Arsenic Trioxide, Thalidomide

## Abstract

**Objective:**

Autophagy and apoptosis play key roles in cancer survival and pathogenesis and are governed by specific genes which have a dual role in both cell death and survival. Arsenic trioxide (ATO) and thalidomide (THAL) are used for treatment of many types of hematologic malignancies. ATO prevents the proliferation of cells and induces apoptosis in some cancer cells. Moreover, THAL has immunomodulatory and antiangiogenic effects in malignant cells. The aim of present study was to examine the effects of ATO and THAL on U937 and KG-1 cells, and evaluation of mRNA expression level of VEGFs genes, PI3K genes and some of autophagy genes.

**Materials and Methods:**

In this *in vitro* experimental study, U937 and KG-1 cells were treated by ATO (0.4-5 µM) and THAL (5-100 µM) for 24, 48 and 72 hours. Cell viability was measured by MTT assay. The apoptosis rate and cell cycle arrest were evaluated by flow cytometry (Annexin/PI) and cell cycle flow cytometry analysis, respectively. The effect of ATO/THAL on mRNAs expression was evaluated by real-time polymerase chain reaction (PCR).

**Results:**

ATO/THAL combination enhanced cell apoptosis in a dose-dependent manner. Also, ATO/THAL induced SubG1/ G1 phase arrest. mRNA expression levels of *VEGFC* (contrary to other VEGFs isoform), *PI3K, AKT, mTOR, MEK1, PTEN, IL6, LC3* and *P62* genes were upregulated in acute myeloid leukemia (AML) cells following treatment with ATO/THAL.

**Conclusion:**

Combined treatment with ATO and THAL can inhibit proliferation and invasion of AML cells by down-regulating *ULK1* and BECLIN1 and up-regulating *PTEN* and *IL6*, and this effect was more marked than the effects of ATO and THAL alone.

## Introduction

**Fig 1:**
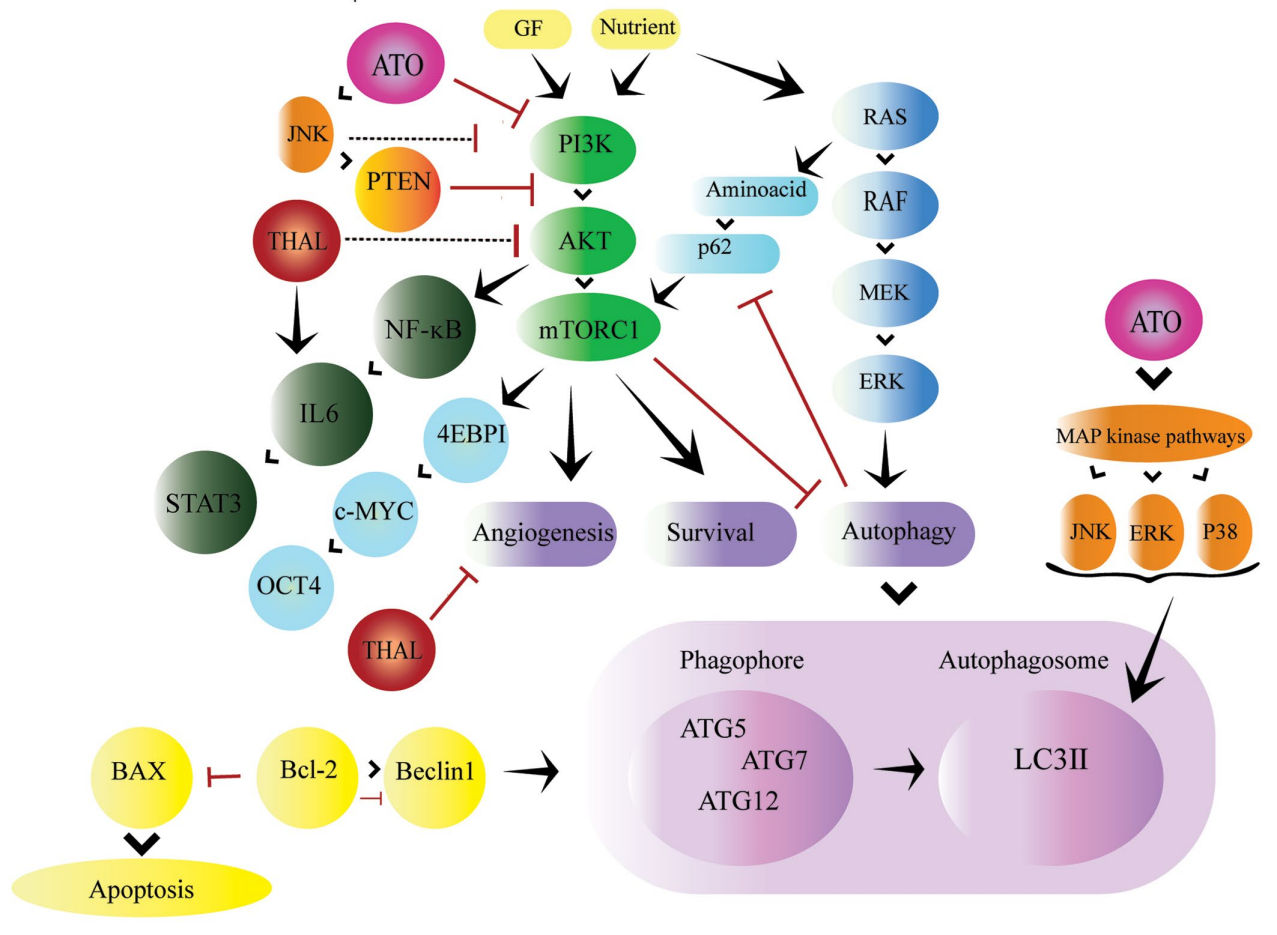
Overview of the PI3K/AKT/mTOR pathway in AML. The PI3K/AKT/mTOR pathway and other pathways related to AML, Inducing autophagy by inhibiting the *mTOR* pathway. Diagram shows that ATO promotes apoptotic mechanisms. Left, PI3K/Akt/NF-κB pathway permanently activated in the absence of ATO. Right, ATO by inducing *JNK* activation, can inhibit the PI3K/Akt/NF-κB signalling pathway. THAL has anti-angiogenesis effects on tumour growth and progression. THAL inhibits *IL6*. ATO/THAL by inhibition of *mTORC1* induces dephosphorylation of *ULK1* and subsequent *ULK1*-mediated phosphorylation of *ATG13, FIP200* and *ULK1* itself, inducing autophagosome synthesis. Release of *BCL-2* suppresses *BECLIN1* that induces autophagy through disruption of the *BCL-2/BCL- XL-BECLIN1* interaction. In case of existence of sufficient nutrients, *BECLIN1* binds to *BCL-2* or *BCL-XL*, and loses its ability to initiate autophagy. AML; Acute myeloid leukemia, ATO; Arsenic trioxide, and THAL; Thalidomide.

Acute myeloid leukemia (AML) is a heterogeneous group of malignancies that is caused by uncontrolled proliferation of neoplastic cells in the bone marrow (1, 2). Approximately 50% of patients ultimately experience relapse after chemotherapy because of the presence of subsets of malignant cells that are not completely removed by treatment regimens (3). Various mechanisms are involved in development of cancers, including alterations in the expression of molecules which impair apoptosis and autophagy (4-6). The PI3K/Akt/ mTOR signalling loop is one of the most important pathways that is deregulated in many human cancers threatening survival of normal cells (6, 7). Hyper activation of the PI3K/Akt/mTOR signalling pathway is an unusual feature of AML patients (7, 8). Phosphatase and tensin homolog (*PTEN*) can negatively regulate the activity of PI3K pathway (9). *PTEN* is a critical negative regulator of PI3K signalling. Raf-MEK1/2-ERK1/2 pathway transmits responses to growth factors and cytokines. Ras/Raf-1/ERK1/2 and PI3K/Akt/mTOR signaling pathways are important regulators of *PTEN* that determines the cellular outcomes of its activation (10). In addition to genes which are involved in apoptosis, autophagy genes play key roles in pathogenesis of cancer.* mTOR* is a central regulator of autophagy with two separate complexes namely, *mTORC1* and *mTORC2. mTORC1* and *PI3K* are negative regulators of autophagy (11) (Fig .1). When autophagy process is initiated, *PI3K* binds to its core units, *BECLIN1* and simplify the usage of autophagy related 5-7-12 (*ATG5-7-12*) on the membranes (phagophores) to form autophagosomes (12).

Arsenic trioxide (ATO) targets various cellular functions through multiple molecular factors (13-15). ATO has numerous biological effects such as apoptotic and anti-proliferative activities (16). Thalidomide (THAL) has immunological effects and anti-angiogenesis effects on tumour growth and progression (17, 18). It was shown that THAL as a *VEGF* inhibitor, in combination with ATO has a synergistic impact on the inhibition of cell proliferation and promotion of apoptosis in AML cell line (19). Hence, the aim of this study was to explore the effect of a combination of ATO and THAL on apoptosis and expression levels of *VEGF* isoforms, *VEGFR1&2, PI3K, AKT, mTOR, PTEN, IL6, STAT3, B-RAF, RAF1, MEK1,* and *B-CL2* and some autophagy genes such as* BECLIN1, LC3-II, ULK1*, and* ATG5-7-12* in leukemic cell lines.

## Materials and Methods

### Reagents

For this *in vitro* experimental study, THAL was purchased from Santa Cruz Company (Texas) and As_2_O_3_ (ATO) was obtained from Sina Darou Company (Iran).5- diphenyltetrazolium bromide (MTT) dye, Annexin V-FITC apoptosis detection kit, dimethyl sulfoxide (DMSO) and diethyl pyro carbonate (DEPC) treated water were purchased from Sigma-Aldrich Company (St. Louis, MO). RPMI 1640 medium and fetal bovine serum (FBS) were obtained from Gibco (Carlsbad, CA). cDNA synthesis kit and SYBR Premix Ex Taq™ were bought from Takara Biotechnology Co (Otsu, Japan).

### Cell lines and cell culture

KG-1 and U937 were purchased from Pasteur Institute )Iran). U937 cells were cultured in RPMI 1640 medium which was supplemented with 10% FBS, 100 μg/mL penicillin and 100 μg/mL streptomycin. KG-1 cells were cultured in RPMI 1640 medium which was supplemented with 20% FBS, 100 μg/mL penicillin and 100 μg/ml streptomycin. Then, cells were incubated at 37˚C in a humidified atmosphere containing 5% CO_2_. THAL was dissolved in DMSO, then dissolved in sterile double-distilled water. As_2_O_3_ was dissolved in distilled water. Each experiment was performed three time in triplicate.

### Analysis of cell viability by MTT assay

KG-1 and U-937 cells (5×10^3^ cells per well) were incubated in the absence or presence of THAL and ATO, in a final volume of 400 μl. After 24, 48 and 72 hours, 100 μl MTT reagent (5 mg/ml MTT in RPMI) was added to each well and incubated for 3 hours. Then, 100 μl DMSO was added to dissolve formazan precipitates. Then, in a 96-well plate (SPL, Life Sciences, Pocheon, Korea), 100 μl of cell lysate were plated in triplicate, and the absorbance was read at 570 nm using an ELISA plate reader (Micro plate Reader; Bio Rad).

### Analysis of cell apoptosis and cell viability by flow cytometry

KG-1 and U937 cells were seeded at the density of 3×10^5^ cells per well in 12-well culture plates then were treated with selective doses, 1.618 μM and 1 μM concentration of ATO respectively in KG-1 and U937 and also 60 μM and 80 μM concentration of THAL in both cell lines. After 48 hours, cells were harvested and treated with Annexin/PI. AnnexinV staining was quantified by FACS Calibur Flow Cytometer analysis (BD-Biosciences, San Jose, CA, USA). Apoptosis (Annexin V+/PI− is early apoptosis and Annexin V+/ PI+ is late apoptosis) and necrosis (Annexin V−/PI+) were investigated in this step.

### Cell cycle analysis

 KG-1 and U937 cells were seeded at the density of 3×105 cells per well in 12-well culture plates then were treated with selective doses, 1.618 μM and 1 μM concentration of ATO respectively in KG-1 and U937 and also 60 μM and 80 μM concentration of THAL in both cell lines. After that, cells fixed in 70% in ethanol and treated with PI. Cells were assessed by BD flow cytometer instrument and results were analyzed by Flowjow software. The apoptotic cells could predict from hypo-diploid sub G1/ G1 DNA content.

### Quantitative real-time polymerase chain reaction

KG-1 and U937 cells were seeded at the density of 5×10^5^ cells per well in 6-well culture plates then were treated with selective doses, 1.618 μM and 1 μM concentration of ATO respectively in KG-1 and U937 cell lines and 80 μM and 60 μM concentration of THAL respectively in KG-1 and U937 cell lines. After that, total RNA was extracted by TriPure Isolation Reagent (Roche applied science, Germany) according to the manufacturer’s instructions. The quality and quantity of total RNA was assessed spectrophotometrically by using Nano Drop ND-1000 (NanoDrop Technologies, Wilmington, DE), and stored at -80˚C. Complementary DNA (cDNA) was manufactured using RNA and cDNA synthesis kit. Real-time RT-PCR analysis was done using a light cycler instrument (Roche Diagnostic, Manheim, Germany) and SYBR Premix Ex Taq. A final volume of 20 μl including 2 μl of a 2-fold diluted cDNA, 10 pmol of primers mixture (0.5 μl of forward and reverse primers), 10 μl of SYBER, and 7 μl of distilled water, was used. PCR reaction included 3 main steps namely, denaturation, annealing and extension. Initial denaturation was done at 94˚C for 5 minutes. After that, denaturation was done at 94˚C for 30 second. In this step, double strands of DNA were separated into two single strands. In the annealing step, the temperature was lowered to enable the DNA primers attach to the template DNA at 50-56˚C for 45 seconds. During the extension, as the final step, the heat was increased to 72˚C to enable the new DNA to be made by a special Taq DNA polymerase enzyme for 1 minute per kb. At the end of PCR reaction, there was final extension at 72˚C for 5 minutes. Data were normalized against *HPRT* expression in each sample. Relative gene expression data were analyzed by 2^-ΔΔCt^ method. Sequences of primers are listed in TableS1 (See Supplementary Online Information at www. celljournal.org).

### Statistical analysis

All experiments were repeated independently at least three times in triplicate, and the data are presented as mean ± SE. The results were compared using standard one-way analysis of variance (ANOVA). The diagrams were generated by GraphPad Prism 6.01 software. Significance was defined as *P<0.05, **P<0.01, and ***P<0.001.

## Results

### ATO and THAL inhibit cell proliferation

**Fig 2:**
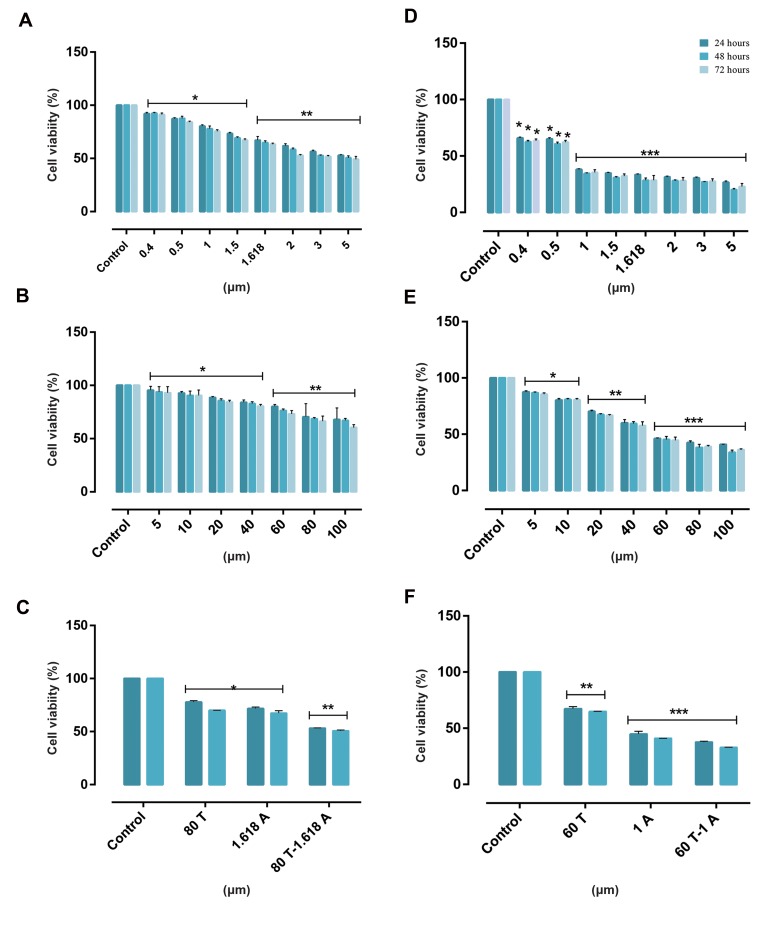
Cell viability in KG-1 and U937. Effects of ATO and THAL on cell viability in KG-1 and U937 cell lines. The anti-proliferative effects of ATO (0.4-5 μM), THAL (5-100 μM) and their combinations in both cell lines were assessed by MTT assay after 24, 48 and 72 hours of treatment. Results obtained following 48 and 72 hours treatment were not significantly different. **A.** Effect of ATO on KG-1 cells, **B.** Effect of THAL on KG-1 cells, **C.** Effect of ATO/THAL on KG-1 cells, **D.** Effect of ATO on U937 cells, **E.** Effect of THAL on U937 cells, and **F.** Effect of ATO/THAL on U937 cells. After detection of suitable doses for ATO (1.618 μM) and THAL (80 μM) for KG-1 and ATO (1 μM) and THAL (60 μM) for U937, effect of a combination of ATO and THAL was evaluated. Data are expressed as mean ± S.E of three independent experiments. Statistical significance was defined at *; P<0.05, **; P<0.01, and ***; P<0.001 compared to corresponding control. ATO; Arsenic trioxide and THAL; Thalidomide.

In KG1 and U937 cell lines, cytotoxic effect of ATO (0.4-5 μM) and THAL (5-100 μM) was investigated. Growth inhibitory effects of these concentrations of ATO/THAL were assessed by MTT for 24, 48 and 72 hours (Fig .2). Based on the results, half maximal inhibitory concentration (IC_50_) values for ATO were 1 μM for U937 cells and 1.618 μM for KG-1 cells; IC_50_ values for THAL were 60 μM for U937 cells and 80 μM for KG-1 cells. The results showed that ATO and THAL had a significant cytotoxic effect on both cell lines in dose- and time-dependent manners. To investigate the synergistic activity of ATO and THAL (80 μM THAL/1.618 μM ATO for KG-1 and 60 μM THAL/1 μM ATO for U937), the viability of treated cells were assessed 24 and 48 and 72 hours post-treatments. Results obtained for 48 and 72 hours treatments were not significantly different. The combination therapy showed a significant effect on U937 and KG-1 cells.

### Induction of apoptosis by ATO/THAL

**Fig 3:**
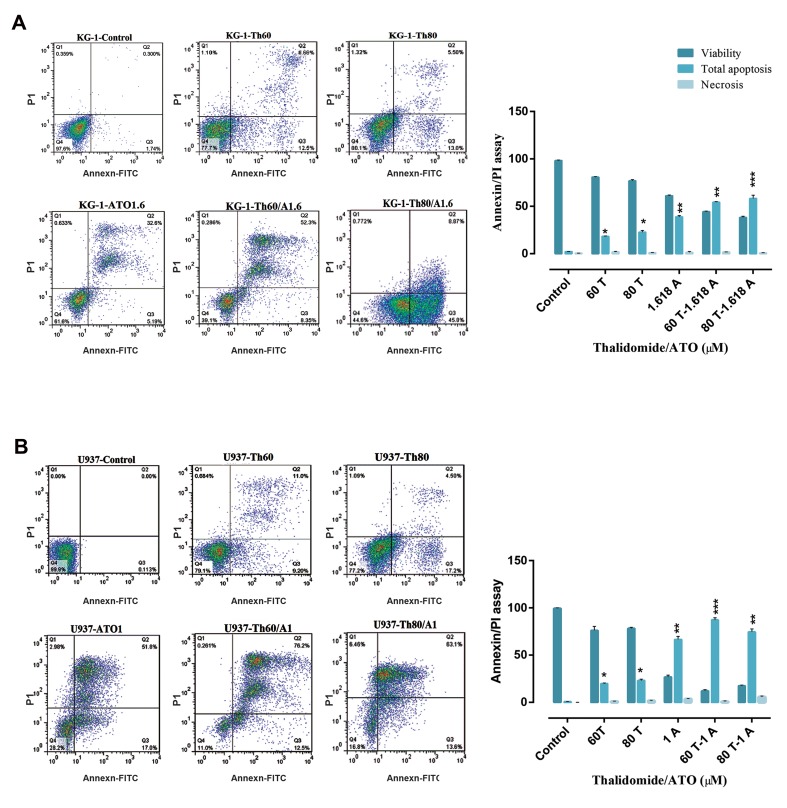
Flow cytometry analysis. **A.** Analysis of flow cytometry in KG-1 cells and **B.** Analysis of flow cytometry in U937 cells. KG-1 cells treated with ATO (1.618 μM) and THAL (80 μM) and their combination. U937 cells treated with ATO (1 μM) and THAL (60 μM) and their combination. Flow cytometry graphs contain the lower left quadrant that shows live cells and the upper left quadrant shows necrotic cells, the lower right shows early apoptotic cells and the upper right shows late apoptotic cells. Data are expressed as mean ± S.E of three independent experiments. Statistical significance was defined at *; P<0.05, **; P<0.01, and ***; P<0.001 compared to corresponding control. ATO; Arsenic trioxide and THAL; Thalidomide.

We performed flow cytometry assay to investigate apoptotic effects of these compounds on AML cell lines. As seen in Figure 3, we observed an increase in the number of early and late apoptotic cells (Annexin+/ PI-+) and minimum percentage of necrosis (Annexin-/ PI+) in treated cells as compared with control in both cell lines. Moreover, significant increases (61% in KG-1 and 88% in U937) in the number of apoptotic cells were seen in cells treated with a combination of ATO and THAL.

### ATO/THAL induces SubG1/G1 arrest in AML cells

**Fig 4:**
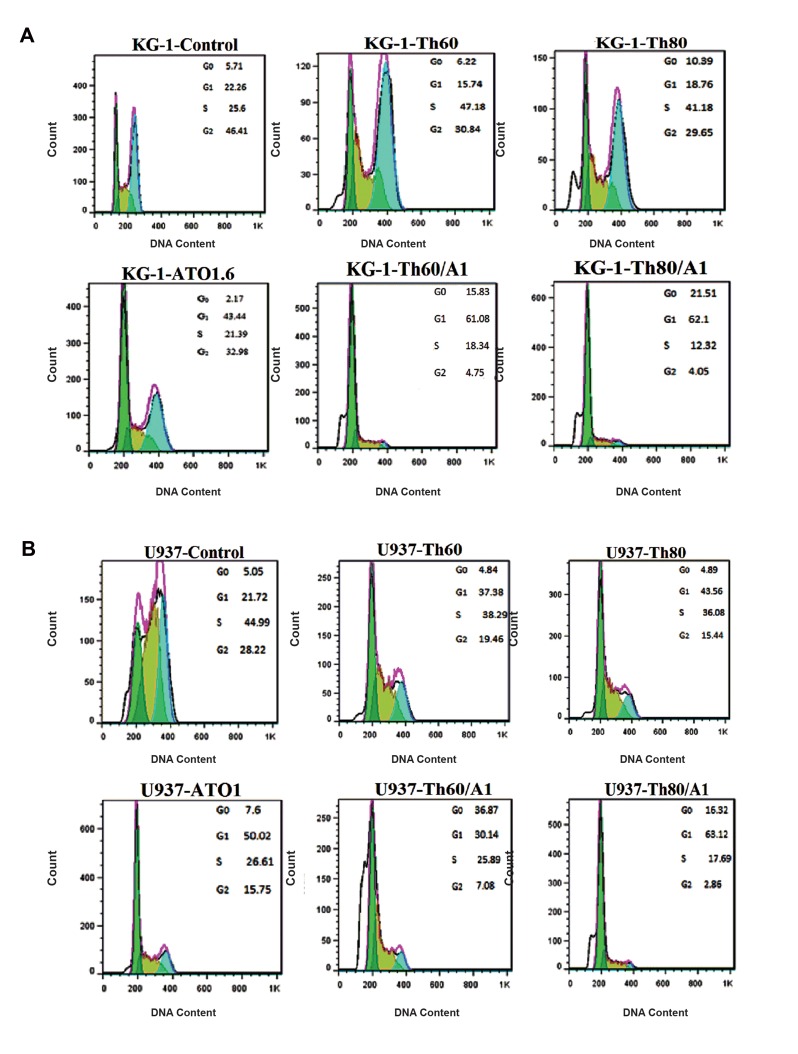
Cell cycle flow cytometry analysis of leukemia cells. **A.** KG-1 cell cycle flow cytometry and **B.** U937 cell cycle flow cytometry. Cells exposed to different concentrations of ATO and THAL for 48 hours, reduced number of cells at G2 phase and increased amount of cells at G1 phase. Data are expressed as mean ± S.E. of three independent experiments. ATO; Arsenic trioxide, and THAL; Thalidomide.

Cell cycle flow cytometry analysis was applied for cells treated with ATO and THAL to study ATO/THAL effects with respect to inducing cell cycle arrest Figure 4 Significant increases in the percentage of cells at SubG1/ G1 were observed in a dose-dependent manner in KG-1 and U937 cells. Meanwhile, percentage of cells at G2 phase was reduced in all treated cells. Accordingly, it seems that ATO/THAL induced SubG1/G1 arrest in both cell lines (5.71-21.51% for KG-1 cell and 5.05-36.87% for U937 cell).

### Real-time polymerase chain reaction

**Fig 5:**
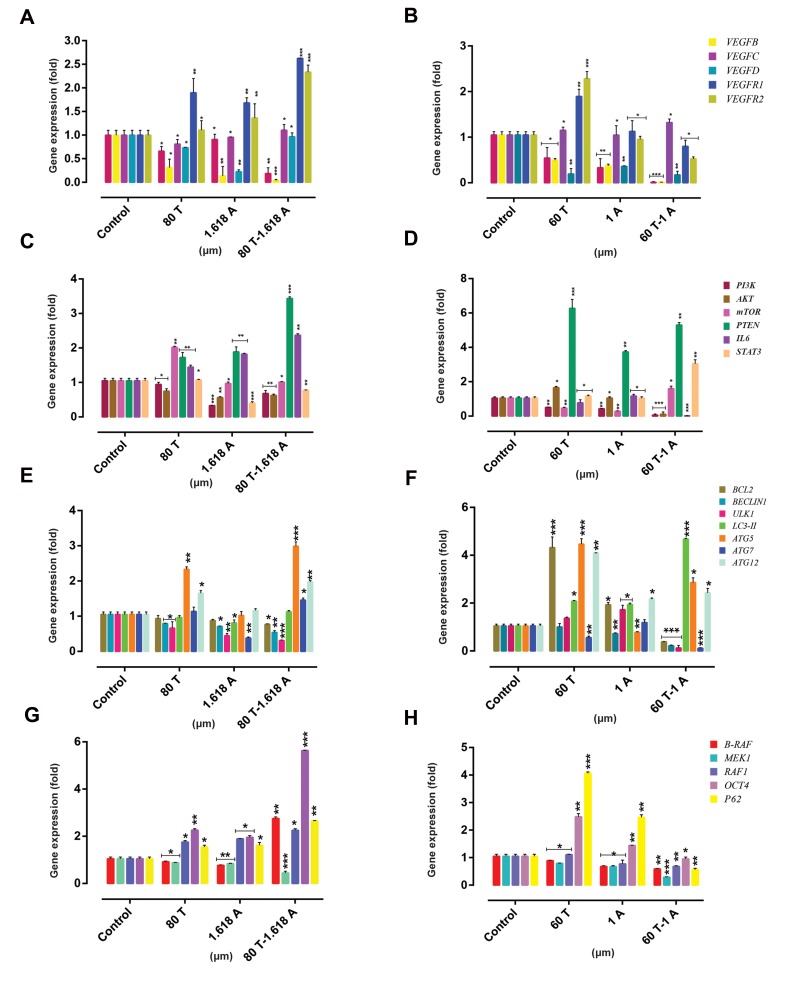
Examination of gene expression. The effects of ATO and THAL on the mRNA level of indicated genes in U937 cells. **A.** Effect of ATO and THAL on the expression level of VEGF genes in KG-1 cells. **B.** Effect of ATO and THAL on the expression level of VEGF genes in U937 cells, **C.** Effect of ATO and THAL on the expression level of genes that contribute to PI3K/AKT/mTOR pathway in KG-1 cells, **D.** Effect of ATO and THAL on the expression level of genes that contribute to PI3K/AKT/mTOR pathway in U937 cells, **E.** Effect of ATO and THAL on the expression level of autophagy genes in KG-1 cells, **F.** Effect of ATO and THAL on the expression level of autophagy genes in U937 cells, **G.** Effect of ATO and THAL on the expression level of BRAF/MEK/RAF1/OCT4/P62 genes in KG-1 cells, and **H.** Effect of ATO and THAL on the expression level of BRAF/MEK/RAF1/OCT4/P62 genes in U937 cells. For normalization of expression levels, HPRT was used. Values are given as mean ± S.E. of three independent experiments. Statistical significance was defined at *; P<0.05, **; P<0.01 and ***; P<0.001 compared to corresponding control. ATO; Arsenic trioxide and THAL; Thalidomide.

We analyzed expression levels of VEGF isoforms and receptors of *VEGF (VEGFR12 &), PI3K, AKT, mTOR, PTEN, IL6, STAT3, MEK1, B-RAF, RAF1, BCL-2, BECLIN1, ULK1, LC3-II, ATG5, ATG7, ATG12, OCT4,* and *P62* by real-time PCR (Fig .5).

U937 cells were treated ATO (1 ìM), THAL (60 ìM) and their combination for 48 hours. We observed that the expression level of *VEGFA* and *VEGFB* significantly decreased and also the expression of VEGFD slightly decreased as compare with *VEGFA/B* when treated with each compound alone or their combination. But expression of *VEGFC* increased when cells were treated with each compound alone or with the combination of both; *VEGFR1* and *VEGFR2* expression increased when cells were treated with each compound alone but decreased when treated with the combination.

The expression level of *PI3K* and downstream genes were also investigated. We observed that the expression of *PI3K* and *IL6* decreased when treated with each compound alone or with the combination of both but *AKT* increased when treated with each compound alone and decreased with the combination of both compounds and *mTOR* expression contrary to *AKT*, decreased when treated with each compound alone and increased with the combination of both compounds and *STAT3* gene expression increased with the combination of both compounds. The expression of *PTEN* as a tumor suppressor, significantly increased after treatment with the combination of both compounds. We observed that the expression of *B-RAF* and *RAF-1* decreased following treatment with selective doses (ATO 1 μM and THAL 60 μM for U937) and their combination (ATO 1 μM /THAL 60 μM). Moreover, in this pathway, the expression of *MEK1* significantly decreased following treatment with the combination of both compounds. Furthermore, the expression of *BCL-2* increased when treated with each compound alone, while significantly decreased following treatment with the combination of both compounds. With respect to autophagy-related genes, we observed that the expression of *ULK1* and *BECLIN1* decreased after treatment while the expression of *LC3-II* increased following treatment. Furthermore, the expression of *ATG5* and *ATG12* increased following treatment with THAL, decreased following treatment with ATO and slightly increased following treatment with the combination of these compounds while the expression of *ATG7* significantly decreased following treatment with the combination of these compounds.

KG-1 cells were treated with ATO (1.618 μM), THAL (80 μM) and also their combination for 48h. Our data indicated that the expression of *VEGFA* and *VEGFB* significantly decreased but *VEGF-C* and *VEGF-D* slightly increased while the expression of *VEGFR1* and *VEGFR2* significantly increased following treatment with each compound alone and their combination. Also the expression of *PI3K* and *AKT* in KG-1 cells decreased and mTOR slightly increased after treatment with cited doses. The expression of *PTEN* as a tumor suppressor significantly increased after treatment with each compound alone and their combination. In addition, *IL6* expression increased with each compound alone and their combination in KG-1 cells. The expression of *STAT3* slightly increased after treatment with the combination of the two compounds.

Expression level of *B-RAF* and *RAF1* increased when treated with each compound alone but *MEK1* decreased. Furthermore, the expression of *BCL-2* slightly decreased. In addition, the expression of *BECLIN1* and *ULK1* as autophagy activator, decreased by each compound alone in KG-1 cells while the expression of *LC3-II* (a marker of the presence of completed autophagosomes) increased. Furthermore, the expression of *ATG5, ATG7,* and *ATG12* increased in combination of two compounds.

## Discussion

The best known regulator of angiogenesis is VEGF, which regulates endothelial proliferation, permeability, and survival (20). Most important member of the VEGF family is *VEGFA* (21). In our previous study, we demonstrated that ATO/THAL downregulates the expression of *VEGFA* and *VEGFB* in KG-1 cell line and downregulates the expression of *VEGFA , VEGFB* and *VEGFD* in U937 cell line (19).

Kruse et al. (22) reported that THAL inhibits angiogenesis by suppression of basic fibroblast growth factor (*bFGF*) and VEGF genes. Keifer et al. (23) reported THAL also inhibits *NF-κB*, a critical regulator of inflammatory processes. Gockel et al. (24) illustrated that THAL induces apoptosis by inhibition of *PI3K-AKT*. In this research, we found that a combination of ATO/THAL significantly reduces the viability of U937 and KG-1 cells while increases cell apoptosis. ATO with anti-leukemic activity in AML cell lines, enhanced the antitumor activity of THAL in both U937 and KG-1 cell population when used in combination.

ATO is used for treatment of many types of hematologic malignancies (25-27). In this study, we observed that cytotoxicity of ATO and induction of apoptosis in both U937 and KG-1 cell lines follow a dose and timedependent pattern. Our results indicated that ATO can influence cell proliferation and cell death pathway. In 2010, Redondo-Muñoz et al. (28) and Goussetis and Platanias (29) in two studies reported that ATO induces cell apoptosis in chronic lymphocytic leukemia (CLL) that involved upregulation of *PTEN* and inhibition of the PI3K/Akt/NF-κB as a survival pathway Goussetis and Platanias (29) also stated that ATO induces upregulation of *PTEN* but downregulation of X-linked inhibitor of apoptosis protein (*XIAP*). Nayak et al. (30) reported that ATO in combination with ATRA leads to decreased activation of *AKT*. Our results showed that ATO inhibits *PI3K/AKT/mTOR* but upregulates *PTEN* gene expression.

Previous study reported that different *MAPK* cascades are activated during treatment of cells with ATO, including *P38, MAP* (31), *JNK* (32), and *ERK* (33). Nayak et al. (30) showed that ATO can promote MAPK pathways as components of a stress response. We studied *B-RAF/ MEK1/RAF1* of *MAPK* signalling for investigating the effect of ATO on this pathway, specially the potential synergistic effects of THAL and ATO on these three central kinases. Goussetis et al reported that ATO can activate autophagy in a leukemic cell population through induction of autophagy process by activation of the *MAPK* pathway (34). We observed that *B-RAF, MEK 1 *and *RAF1* expression was increased by ATO and THAL and their combination in KG-1 cell line. In addition, the expression level of *MEK1*, and *RAF1* decreased following treatment of U937 cell line with a combination of the compounds.

ATO prevents cell proliferation and induces apoptosis in some cancer cells. Recently, some reports showed the effects of ATO on autophagy (35-37). ATO is a powerful inducer of autophagy in acute leukemia cells. ATO by inhibition of *mTOR*, can induce autophagy. Moreover, activation of *MAPK* pathway by ATO can induce autophagy pathway. Verma et al. (31) showed that ATO also induces autophagy in APL cells. Autophagy is mainly controlled by *mTOR* (11). The *mTOR* acts as a suppressor of autophagy in response to nutrient and growth-factor accessibility. Karantza-Wadsworth et al. (38) and Boya et al. (39) showed that autophagy can increase apoptosis in cancer cells. Kruse et al. (22) illustrated that ATO up regulates *LC3-II* in KT1 cells. Chiu et al. (40) reported that ATO treatment increases the expression of LC3-II, p62, Beclin 1, Atg5, and Atg5-12 proteins.

In the present study, we investigated the expression of *Beclin1, LC3-II, ULK1* and *ATG5-7-12* as autophagy activators. Expression level of *BECLIN1* and *ULK1* decreased in U937 cell line whereas the expression of *LC3-II* increased following treatment with a combination of the compounds.

One of the important gene associated with apoptosis is *BCL-2*, which is a suppressor of programmed cell death. The expression of *BCL-2* declined following ATO treatment in U118-MG cells (40). Our results showed that the level of *BCL-2* gene decreases following treatment with a combination of ATO and THAL.

## Conclusion

This study demonstrated that ATO in combination with THAL promotes apoptotic mechanisms and by inhibition of PI3K/Akt/mTOR signalling pathway, promotes autophagy in AML cells. These findings implied that ATO/THAL may be used as a novel therapeutic agent for inhibition of AML cells.

## Supplementary PDF


