# Supplementary material for: Arsenic Trioxide and Thalidomide Combination Induces Autophagy Along with Apoptosis in Acute Myeloid Cell Lines
Source: Cell J. 2019 Oct 14;22(2):193–202. doi: 10.22074/cellj.2020.6469 (PMC6874786; doi:10.22074/cellj.2020.6469)
Supplement: Supplementary file 1 [file Cell-J-22-193-s01.pdf]

www.celljournal.org

Supplementary Information for

Arsenic Trioxide and Thalidomide Combination Induces Autophagy Along with Apoptosis in Acute Myeloid Cell Lines

Mahnaz Mohammadi Kian, M.Sc.[1, 2], Atousa Haghi, M.Sc.[1, 3], Mahdieh Salami, M.Sc.[1, 2], Bahram Chahardouli, Ph.D.[1, 2], Shahrbanoo Rostami, Ph.D.[1, 2], Kianoosh Malekzadeh, Ph.D.[4], Hosein Kamranzadeh Foumani, M.D.[1, 2], Saeed Mohammadi, Ph.D.[1, 2]*, Mohsen Nikbakht, Ph.D.[1, 2]*

1. Hematology, Oncology and Stem Cell Transplantation Research Center, Tehran University of Medical Sciences, Tehran, Iran
2. Hematologic Malignancies Research Center, Tehran University of Medical Sciences, Tehran, Iran
3. Young Researchers and Elite Club, Pharmaceutical Sciences Branch, Islamic Azad University, Tehran, Iran
4. Molecular Medicine Research Center (MMRC), Hormozgan University of Medical Science (HUMS), Bandar Abbass, Iran

**Corresponding Address: P.O.Box: 1411713131, Hematology, Oncology and Stem Cell Transplantation Research Center, Tehran University of Medical Sciences, Tehran, Iran*
Emails: m-nikbakht@sina.tums.ac.ir, smohammadi@sina.tums.ac.ir

Table S1: Sequences of primers

Genes	Primer sequences (5´- 3´)	Amplicon length (bp)	T_a (°C)	Ref
GAPDH	F:TGAACGGGAAGCTCACTGG<br>R:TCCACCACCCTGTTGCTGTA	307	60	(1)
HPRT	F:GCTATAAATTCTTTGCTGACCTGCTG<br>R:AATTACTTTTATGTCCCCTGTTGACTGG	140	62	(2)
VEGFA	F:AGGGCAGAATCATCACGAAGT<br>R:AGGGTCTCGATTGGATGGCA	75	61	(3)
VEGFB	F:GAGATGTCCCTGGAAGAACACA<br>R:GAGTGGGATGGGTGATGTCAG	172	62	(3)
VEGFC	F:GAGGAGCAGTTACGGTCTGTG<br>R:TCCTTTCCTTAGCTGACACTTGT	96	61	(3)
VEGFD	F:GTATGGACTCTCGCTCAGCAT<br>R:AGGCTCTCTTCATTGCAACAG	226	60	(3)
VEGFR1	F:CAGGCCCAGTTTCTGCCATT<br>R:TTCCAGCTCAGCGTGGTCGTA	82	63	(4)
VEGFR2	F:CCAGCAAAAGCAGGGAGTCTGT<br>R:TGTCTGTGTCATCGGAGTGATATCC	87	63	(5)
PI3K	F:TGCTATGCCTGCTCTGTAGTGGT<br>R:GTGTGACATTGAGGGAGTCGTTG	175	63	(6)
AKT	F:GTGCTGGAGGACAATGACTACGG<br>R:AGCAGCCCTGAAAGCAAGGA	194	63	(6)
mTOR	F:TCCTGAAGAACATGTGCGAG<br>R:CCAAAGTACAAGCGAGAGGC	139	59	(6)
IL6	F:AGTAGTGAGGAACAAGCCAGA<br>R:TACATTTGCCGAAGAGCC	236	58	(7)
STAT3	F:TGGCACCTTGGATTGAGAGTC<br>R:GCAGGAATCGGCTATATTGCT	117	60	(7)
LC3-II	F:GATGTCCGACTTATTCGAGAGC<br>R:TTGAGCTGTAAGCGCCTTCTA	167	60	(8)
Beclin1	F:AGCTGCCGTTATACTGTTCTG<br>R:ACTGCCTCCTGTGTCTTCAATCTT	185	62	(9)
ULK1	F:TCGAGTTCTCCCGCAAGG<br>R:CGTCTGAGACTTGGCGAGGT	134	61	(10)
BCL-2	F:CTGCACCTGACGCCCTTCACC<br>R:CACATGACCCCACCGAACTCAAAGA	119	65	(11)>
PTEN	F:TGGATTCGACTTAGACTTGACCT<br>R:TTTGGCGGTGTCATAATGTCTT	139	59	(12)
B-RAF	F:CTCGAGTGATGATTGGGAGATTCCTGATGG<br>R:CTGCTGAGGTGTAGGTGCTGTCAC	148	66	(13)
RAF-1	F:CAG CCC TGT CCA GTA GC<br>R:GCG TGA CTT TAC TGT TGC	614	57	(13)
MEK1	F:ACCAGCCCAGCACACCAA<br>R:GGGACTCGCTCTTTGTTGCTT	68	61	(14)

References

1. Kong X, Xu X, Yan Y, Guo F, Li J, Hu Y, et al. Estrogen regulates the tumour suppressor MiRNA-30c and its target gene, MTA-1, in endometrial cancer. PLoS One. 2014; 9(3): e90810.
2. Gusenbauer S, Zanucco E, Knyazev P, Ullrich A. Erk2 but not Erk1 regulates crosstalk between Met and EGFR in squamous cell carcinoma cell lines. Mol Cancer. 2015; 14: 54.
3. Mohammadi Kian M, Mohammadi S, Tavallaei M, Chahardouli B, Rostami S, Zahedpanah M, et al. Inhibitory Effects of Arsenic Trioxide and Thalidomide on Angiogenesis and Vascular Endothelial Growth Factor Expression in Leukemia Cells. Asian Pac J Cancer Prev. 2018; 19(4): 1127-1134.
4. Grellier M, Ferreira-Tojais N, Bourget C, Bareille R, Guillemot F, Amédée J. Role of vascular endothelial growth factor in the communication between human osteoprogenitors and endothelial cells. J Cell Biochem. 2009; 106(3): 390-398.
5. Giurdanella G, Anfuso CD, Olivieri M, Lupo G, Caporarello N, Eandi CM, et al. Aflibercept, bevacizumab and ranibizumab prevent glucose-induced damage in human retinal pericytes in vitro, through a PLA2/COX-2/VEGF-A pathway. Biochem Pharmacol. 2015; 96(3): 278-287.
6. Guo JR, Wang H, Jin XJ, Jia DL, Zhou X, Tao Q. Effect and mechanism of inhibition of PI3K/Akt/mTOR signal pathway on chronic neuropathic pain and spinal microglia in a rat model of chronic constriction injury. Oncotarget. 2017; 8(32): 52923-52934.
7. Wang X, Li Y, Dai Y, Liu Q, Ning S, Liu J, et al. Sulforaphane improves chemotherapy efficacy by targeting cancer stem cell-like properties via the miR-124/IL-6R/STAT3 axis. Sci Rep. 2016; 6: 36796.
8. Zhao Y, Yang J, Liao W, Liu X, Zhang H, Wang S, et al. Cytosolic FoxO1 is essential for the induction of autophagy and tumour suppressor activity. Nat Cell Biol. 2010; 12(7): 665-675.
9. Wang J, Pan XL, Ding LJ, Liu DY, Da-Peng Lei, Jin T. Aberrant expression of Beclin-1 and LC3 correlates with poor prognosis of human hypopharyngeal squamous cell carcinoma. PLoS One. 2013; 8(7): e69038.
10. Gao W, Shen Z, Shang L, Wang X. Upregulation of human autophagy-initiation kinase ULK1 by tumor suppressor p53 contributes to DNA-damage-induced cell death. Cell Death Differ. 2011; 18(10): 1598-1607.
11. Cianfrocca R, Tocci P, Semprucci E, Spinella F, Di Castro V, Bagnato A, et al. β-Arrestin 1 is required for endothelin-1-induced NF-κB activation in ovarian cancer cells. Life Sci. 2014; 118(2): 179-184.
12. Mohammadi S, Ghaffari SH, Shaiegan M, Zarif MN, Nikbakht M, Akbari Birgani S, et al. Acquired expression of osteopontin selectively promotes enrichment of leukemia stem cells through AKT/mTOR/PTEN/β-catenin pathways in AML cells. Life Sci. 2016; 152: 190-198.
13. Gronych J, Korshunov A, Bageritz J, Milde T, Jugold M, Hambardzumyan D, et al. An activated mutant BRAF kinase domain is sufficient to induce pilocytic astrocytoma in mice. J Clin Invest. 2011; 121(4): 1344-1348.
14. Lin DA, Boyce JA. IL-4 regulates MEK expression required for lysophosphatidic acid-mediated chemokine generation by human mast cells. J Immunol. 2005; 175(8): 5430-5438.
